# Post-Coronavirus Disease-2019 (COVID-19): Toward a Severe Multi-Level Health Crisis?

**DOI:** 10.3390/medsci9040068

**Published:** 2021-11-08

**Authors:** Abdelaziz Ghanemi, Mayumi Yoshioka, Jonny St-Amand

**Affiliations:** 1 Functional Genomics Laboratory, Endocrinology and Nephrology Axis, CREMI, CHU de Québec-Université Laval Research Center, 2705 Boul. Laurier, Quebec City, QC G1V 4G2, Canada; Abdelaziz.Ghanemi@crchudequebec.ulaval.ca (A.G.); mayumi.yoshioka@crchudequebec.ulaval.ca (M.Y.); 2 Department of Molecular Medicine, Faculty of Medicine, Laval University, Quebec City, QC G1V 0A6, Canada

**Keywords:** coronavirus disease-2019 (COVID-19), post-crisis, health

## Abstract

There were already numerous challenges facing the healthcare system prior to the ongoing coronavirus disease-2019 (COVID-19) pandemic. Although we look forward to ending this pandemic, it is still expected that the healthcare system will face further challenges leading to a multi-level health crisis. Indeed, after the COVID-19 pandemic, there will still be COVID-19 active cases and those left with health problems following COVID-19 infection who will be of a particular impact. In addition, we also have the health problems that either emerged or worsened during COVID-19, especially with the reduced ability of the healthcare system to take care of many non COVID-19 patients during the COVID-19 pandemic. Such expected evolution of the situation highlights the necessity for the decision-makers to consider applying serious reforms and take quick measures to prevent a post-COVID-19 health crisis.

The coronavirus disease-2019 (COVID-19) pandemic has put intense pressure on the health system, especially during the periods of “waves” [1,2]. The reduction of COVID-19 crisis evolution indicators (mainly daily cases and mortality), resulting from the combination of both the applied measures (mainly lockdown, masks and physical distancing) [3,4] and the vaccination [5], seems to point toward an end of this crisis (at least the most critical phases) [6]. However, even after the COVID-19 crisis passes, the healthcare system will remain under pressure as a result of a variety of consequences induced, directly and indirectly, by the COVID-19 crisis. The upcoming crises would have four main origins via which it puts pressure on the health care system and health professionals.

**COVID-19 active cases and those left with health problems following COVID-19 infection are of a particular impact**. Indeed, even after the COVID-19 pandemic is under control, numerous COVID-19 patients could require a long-term care. This is due to the fact that COVID-19 can leave the patient with health problems even after the patient tests negative (cured), such as sleep difficulties, anxiety, depression and severely impaired pulmonary diffusion capacities [7]. This is important knowledge due to the millions of COVID-19 cases being registered in over 200 countries [8,9]. Each one of these health consequences would require therapies and follow-ups with healthcare professionals along with the associated financial and social consequences. Post-intensive care syndrome (PICS) represents an illustrative example of the post-COVID-19 pandemic health consequences. PICS has been defined as a new clinical entity [10] in which intensive care unit survivors have health problems, including mental health problems, prolonged physical impairments and cognitive impairments [11]. The patients would require a follow-up by cognitive, physical and psychiatric specialists [12] as the symptoms they suffer from could persist for years [13], resulting in health and economic challenges [14]. Importantly, these physical and mental health problems can impact the lifestyle and lead, for instance, to reduced physical activity and unhealthy dietary and sleeping habits. Such an unbalanced lifestyle may lead to other pathologies, including obesity, cardiovascular diseases and metabolic disorders, and worsen mental health as well.

**Another pathological aspect is related to the diseases that emerged or worsened during COVID-19**. During the crisis and with the imposed measures along with the newly acquired habits, numerous health problems have seen their epidemiological profiles or risks worsened. This includes obesity (due to confinement and a reduced physical activity) [15,16,17], immunity decline [18], sarcopenia [19] and mental health [20,21,22] as well as an unhealthy lifestyle (diet, sleep, etc.), that impact the metabolic profile, which represents a risk factor for diabetes, hypertension and cardiovascular diseases [23]. Such health problems also increase the vulnerability to other diseases. For instance, while reduced immunity could increase exposure to numerous infectious diseases and reduce vaccination efficacy [18], obesity is a risk factor for diseases, including diabetes, cardiovascular diseases, perturbed immunity, regeneration impairment and metabolic disorders [24,25,26,27,28,29,30,31,32,33,34,35,36]. Moreover, the diseases and health problems resulting from COVID-19, including pneumonia and acute respiratory distress syndrome [37,38], further worsen this situation, especially if the infected patients are among those already suffering from other diseases, including cardiovascular diseases, HIV and diabetes [39]. This puts further pressure on the health care system and worsens the health crises.

The COVID-19 crisis has not only generated novel challenges in terms of worsening the health profile of the population, but it **has reduced the ability of the health care system to take care of many non-COVID-19 patients**. Indeed, most hospitals that have been submerged by COVID-19 patients have had to concentrate their efforts on treating COVID-19 patients. This has resulted in delaying non-urgent healthcare of non-COVID-19 patients, including elective surgeries [40]. In addition, many individuals delayed visiting or were not able to see a healthcare professional or they simply did not seek health care because of the psychological phobia (corona-phobia [41]) of visiting hospitals during the pandemic. Within this context, the closed borders and the limitation of inter-regional travel [42,43] also impacted health care access for those who failed to find the required therapies in their regions and needed to travel to get them. This resulted in delays in diagnosis as well as treatment, which may lead to poor prognosis, especially for diseases that evolve quickly, such as cancer [44,45]. All these factors lead to delays in treating cases that require urgent care. This situation represents a challenge for the “post-COVID-19 crisis” that includes COVID-19 patients and those suffering from health problems resulting either indirectly from COVID-19 infections or problems resulting from the implemented measures, further worsening the public health pattern.

Finally, in addition to the above challenges, **the regular health care system has** (similar to what we had prior to COVID-19) **already experienced challenges** and was in need of reform prior to the COVID-19 crisis. Now, such need is more urgent, especially since many healthcare workers left their jobs permanently or temporarily either as a decision (to quit for example), because they were too vulnerable to continue working with COVID-19 risk (pregnancy, immune-depression, etc.) or had to leave because they tested positive for COVID-19 or due to mental health issues [46]. This situation impacts the health care system capacity both during and after the COVID-19 crisis.

All these factors (Figure 1) make the upcoming period critical for the healthcare system. We are heading toward a phase with an increased pathological and poor public health pattern with a multi-level crisis involving health, the economy and society. This makes finding innovative solutions urgent, especially as another pandemic is always a possibility. Such solutions cannot be limited to healthcare system reform but must include population education within a multidisciplinary approach aiming both to improve the healthcare performance and the public health profile. We can even be inspired by the “positive outcomes” of the COVID-19 crisis [47] in order to improve public health.

Even before the COVID-19 pandemic, the healthcare system was facing diverse challenges. After the COVID-19 pandemic, there will still be various challenges for the healthcare system. COVID-19 active cases and those left with health problems following COVID-19 infection (such as the post-intensive care syndrome) are of a particular impact. In addition, we have the health problems that will emerge or worsen during COVID-19 because of the reduced healthcare for non-COVID-19 patients.

## Figures and Tables

**Figure 1:**
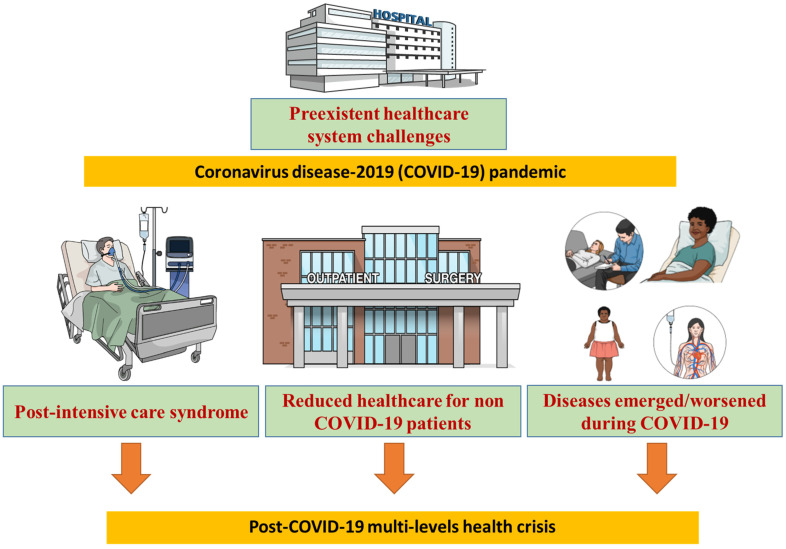
Post-coronavirus disease-2019 (COVID-19) multi-levels health crisis.

## Data Availability

Not applicable.
